# Nasal NUT carcinoma with repeated responses during multimodal treatment incorporating radiotherapy: A case report

**DOI:** 10.1097/MD.0000000000050248

**Published:** 2026-09-18

**Authors:** Qi An, Yuxuan Tao, Peiguo Wang, Zhongqiu Wang

**Affiliations:** a Department of Radiation Oncology, Tianjin Medical University Cancer Institute & Hospital, National Clinical Research Center for Cancer, Tianjin’s Clinical Research Center for Cancer, Laboratory of Basic and Translational Medicine on Head & Neck Cancer, Key Laboratory of Cancer Prevention and Therapy, Tianjin, China.

**Keywords:** case report, multimodal treatment, nasal cavity, NUT carcinoma, radiotherapy

## Abstract

**Rationale:**

Nuclear protein in testis (NUT) carcinoma, formerly referred to as NUT midline carcinoma, is an exceptionally rare and aggressive malignancy. Evidence guiding radiotherapy-based management is limited, especially for nasal primary tumors with neuroaxis and systemic dissemination.

**Patient concerns:**

A 40-year-old woman was referred after resection of a nasal cavity malignancy. During the subsequent course, she experienced orbital pain, headache, visual impairment, severe lumbar and radicular pain, bilateral lower-limb paralysis, urinary retention, malignant pleural effusion, and widespread metastatic disease.

**Diagnoses:**

Pathologic consultation supported nasal NUT carcinoma, with positive NUT immunostaining and a Ki-67 index of approximately 70%. External molecular testing was reported to confirm a NUTM1 rearrangement, although the original report could not be obtained for review, and the assay platform and fusion partner could not be independently verified.

**Interventions:**

After surgery, the patient received postoperative VMAT/IMRT to 70 Gy in 35 fractions with concurrent cisplatin. Following leptomeningeal, cauda equina, and extensive osseous dissemination, she received palliative helical IMRT/Tomotherapy craniospinal irradiation to 15 Gy in 10 fractions. A later VMAT plan delivered 30 Gy in 10 fractions to one lumbar and 2 hepatic targets. Pembrolizumab, bevacizumab, temozolomide, pleural drainage, and intrapleural therapy overlapped with different treatment phases.

**Outcomes:**

During the first course, headache severity decreased from 8/10 to 2.5/10 on a visual analog scale, and clinically recorded Snellen visual acuity improved from 20/200 to 20/50. Pain relief was documented after craniospinal irradiation and after the third treatment course. Serial imaging was contemporaneously interpreted as indicating interval reduction in selected lesions after radiation-containing multimodal treatment phases, although uniform retrospective remeasurement was not feasible. According to telephone follow-up with the patient’s family, the patient died approximately 12 months after surgery.

**Lessons:**

Repeated clinical and imaging responses were observed following multiple phases of multimodal treatment incorporating radiotherapy. Because systemic therapies were administered during overlapping periods, the independent contribution of radiotherapy could not be isolated. Radiotherapy may provide clinically meaningful local or palliative benefit as part of individualized multimodal treatment in selected patients.

## 1. Introduction

Nuclear protein in testis (NUT) carcinoma, formerly referred to as NUT midline carcinoma, is a rare, aggressive malignancy defined by rearrangement of NUTM1 and aberrant nuclear NUT expression. It frequently arises in midline structures, including the head and neck, and is associated with rapid progression and poor survival.^[1,2]^

Because of its rarity, evidence is derived mainly from retrospective series, pooled analyses, and case reports. Surgery, radiotherapy, chemotherapy, immunotherapy, and targeted agents have been used in heterogeneous combinations, but no universally accepted standard of care has been established.^[3–6]^ Intensive local treatment may benefit selected patients, yet dissemination can occur rapidly despite multimodal treatment.

We report a patient with nasal NUT carcinoma in whom repeated clinical and imaging responses were observed after several multimodal treatment phases incorporating radiotherapy: postoperative chemoradiotherapy, palliative craniospinal irradiation (CSI), and palliative irradiation of lumbar and hepatic metastatic targets. The observation is presented as evidence of possible local or palliative benefit within multimodal care, not as proof of intrinsic radiosensitivity, durable control, or survival benefit.

## 2. Case presentation

### 2.1. Patient information

A 40-year-old woman underwent endoscopic surgery at an outside hospital on August 29, 2023, for a nasal cavity and paranasal sinus malignancy. The external pathologic diagnosis was poorly differentiated carcinoma. After surgery, she developed progressive visual impairment, visual-field loss, bilateral orbital pain (left greater than right), and loss of smell. She was referred to our institution for pathologic review and postoperative management.

### 2.2. Diagnostic assessment

Expert pathologic consultation supported NUT carcinoma on the basis of morphology and immunohistochemistry (IHC), including positive NUT staining, a Ki-67 index of approximately 70%, partial cytokeratin expression, focal CD56 expression, retained INI1 and BRG1 expression, and a programmed death-ligand 1 combined positive score of 1. The complete structured findings are provided in Table 1. Tumor profiling was reported to identify a PIK3CA missense variant (c.571A > G; p.I191V).

**Table 1:** Immunohistochemical and molecular findings.

Marker/test	Result	Method/source	Diagnostic relevance
NUT	Positive	Immunohistochemistry reported in pathology consultation	Supports NUT carcinoma in the appropriate morphologic context
Ki-67	Approximately 70%	Immunohistochemistry	High proliferative index
CKpan; CK; CK5/6	Partially positive	Immunohistochemistry	Supports epithelial differentiation
CD56	Focally positive	Immunohistochemistry	Nonspecific supporting finding
INI1; BRG1	Retained/positive	Immunohistochemistry	Retained expression
CD3; CD20; synaptophysin; P40; S-100; CK7; VIM; TTF-1	Negative	Immunohistochemistry	Assists differential diagnosis
EGFR; VEGF	EGFR focally positive; VEGF positive	Immunohistochemistry	Reported tumor biomarker expression
PD-L1	CPS = 1	Pathology record; assay details unavailable	Reported biomarker result; its predictive significance in NUT carcinoma remains uncertain.
PIK3CA	c.571A > G; p.I191V	Tumor molecular profiling; platform details unavailable	Molecular finding of uncertain case-specific therapeutic relevance
NUTM1 rearrangement	Reported positive	External molecular testing; original report unavailable for review	Reported molecular confirmation of NUT carcinoma; the assay platform and fusion partner could not be independently verified

CPS = combined positive score, EGFR = epidermal growth factor receptor, NUT = nuclear protein in testis, PD-L1 = programmed death-ligand 1, VEGF = vascular endothelial growth factor.

External molecular testing was reported to confirm a NUTM1 rearrangement. However, the original molecular report could not be obtained for review; therefore, the specific assay platform and fusion partner could not be independently verified.

### 2.3. Timeline

The major clinical events, diagnostic assessments, systemic treatments, radiotherapy courses, and outcomes are summarized chronologically in Table 2.

**Table 2:** Timeline of the clinical course.

Date/time	Clinical event	Diagnostic findings	Systemic treatment	Radiotherapy	Clinical or imaging outcome
2023-08-29	Endoscopic surgery at an outside hospital	External pathology initially favored poorly differentiated carcinoma; later consultation supported NUT carcinoma	None documented	None	Postoperative recovery; subsequent orbital pain and visual deterioration
2023-11-03 to 2023-12-22	Postoperative local-regional treatment	CTV included postoperative sinonasal/skull-base-related regions and bilateral neck drainage; PTV was a 3-mm expansion	Cisplatin 130 mg, divided over 2 d, on 2023-11-07 and 2023-11-28	VMAT/IMRT, final prescription 70 Gy/35 fractions	VAS headache score decreased from 8/10 to 2.5/10 and clinically recorded Snellen visual acuity improved from 20/200 to 20/50 during treatment; later new systemic symptoms developed
2024-01-04 and 2024-01-08	Neuroaxis and pelvic reassessment	MRI showed leptomeningeal disease involving the filum terminale and cauda equina and multifocal osseous disease involving the lumbar spine, sacrum, bilateral ilia, acetabular regions, and left pubis	None documented on imaging dates	None	Severe lumbar/radicular pain, lower abdominal pain, paralysis, and urinary retention
2024-01-09	Systemic treatment initiated for disseminated disease	Disseminated neuroaxis and osseous disease	Pembrolizumab 200 mg	None	Systemic treatment overlapped the subsequent CSI period
2024-01-18 to 2024-01-31	Palliative CSI	PTV approximately 2092.47 cm^3^; mean dose approximately 15.68 Gy; coverage approximately 99.99%	Pembrolizumab preceded CSI; bevacizumab and temozolomide were used subsequently	Helical IMRT/Tomotherapy, 15 Gy/10 fractions	Pain decreased; bilateral lower-limb paralysis persisted
2024-02-19 and 2024-02-20	Ongoing systemic treatment	Persistent disseminated disease and paraplegia	Bevacizumab 500 mg, then pembrolizumab 200 mg	None	Supportive care continued
2024-03-05 to 2024-03-18	Sinonasal and skull-base MRI and evaluation of new dyspnea	Sinonasal and skull-base MRI demonstrated post-treatment changes with indeterminate abnormalities involving the clivus and posterior nasopharyngeal wall. CT showed right pleural effusion, a left lung nodule (approximately 12 × 10 mm), and multiple liver lesions (representative lesion approximately 35 × 29 mm); pleural fluid contained malignant cells	Intrapleural cisplatin 30 mg plus bevacizumab 300 mg on 2024-03-18	None	Dyspnea improved after drainage; improvement cannot be attributed to radiotherapy
2024-03-26 to 2024-04-24	Maintenance systemic therapy	Persistent pleural, pulmonary, hepatic, and osseous metastatic disease	Bevacizumab 500 mg on 2024-03-26 and 2024-04-24; pembrolizumab 200 mg on 2024-04-02 and 2024-04-23; oral temozolomide during this period	None	Progressive lumbosacral pain prompted repeat MRI; multifocal osseous metastatic disease persisted.
2024-04-22	MRI reassessment in the setting of progressive lumbosacral pain	Multifocal abnormalities involving the lumbar spine, sacrum, bilateral ilia, acetabular regions, and right ischium	Ongoing systemic therapy	Planning for third course	Progressive lumbosacral pain
2024-04-28 to 2024-05-13	Third radiation-containing treatment phase	One lumbar target and 2 hepatic targets were treated in one plan	Bevacizumab, pembrolizumab, and temozolomide overlapped nearby periods; pleural drainage was repeated for recurrent effusion	VMAT, 30 Gy/10 fractions to each target	Lumbar pain decreased; dyspnea improved after pleural drainage, not ascribed to radiotherapy
Approximately 12 mo after surgery	Telephone follow-up with family	No formal death certificate was available for review	Not available	None	The family reported that the patient had died

CSI = craniospinal irradiation, CT = computed tomography, CTV = clinical target volume, MRI = magnetic resonance imaging, PTV = planning target volume, VAS = visual analog scale, VMAT = volumetric modulated arc therapy.

Details of the 3 radiotherapy courses are provided in Table 3.

**Table 3:** Details of the 3 radiotherapy courses.

Course	Indication	Treatment intent	Technique	Target volume	Dose/fractionation	Concurrent or overlapping systemic therapy	Documented outcome	Interpretation limitation
1 (2023-11-03 to 2023-12-22)	Postoperative local-regional management	Postoperative treatment	VMAT/IMRT	Postoperative sinonasal and skull-base-related CTV with bilateral neck drainage; PTV = CTV + 3 mm	70 Gy/35 fractions	Cisplatin 130 mg divided over 2 days for 2 cycles	VAS headache score 8/10 to 2.5/10; clinically recorded Snellen visual acuity 20/200 to 20/50	Concurrent cisplatin prevents isolation of the radiotherapy contribution; no formal ophthalmologic examination report was available
2 (2024-01-18 to 2024-01-31)	Leptomeningeal, filum terminale, cauda equina, and extensive osseous disease	Palliative CSI	Helical IMRT/Tomotherapy	Craniospinal PTV, approximately 2092.47 cm^3^	15 Gy/10 fractions	Pembrolizumab preceded treatment; bevacizumab and temozolomide followed/overlapped nearby	Pain decreased; paralysis persisted	Low palliative dose selected to balance symptom relief, treatment burden, and normal-tissue exposure in rapidly progressive disseminated disease
3 (2024-04-28 to 2024-05-13)	Progressive lumbar pain and hepatic metastatic targets	Palliative	VMAT	One lumbar metastatic target and 2 hepatic metastatic targets in one plan	30 Gy/10 fractions to each target	Bevacizumab, pembrolizumab, and temozolomide during nearby/overlapping periods	Lumbar pain decreased; contemporaneous imaging was interpreted by the treating team as indicating treatment response.	Systemic therapies overlapped this treatment phase; therefore, the independent contribution of radiotherapy could not be isolated. Improvement in dyspnea followed pleural drainage and was not attributed to radiotherapy.

CSI = craniospinal irradiation, CTV = clinical target volume, IMRT = intensity-modulated radiotherapy, PTV = planning target volume, VAS = visual analog scale, VMAT = volumetric modulated arc therapy.

### 2.4. Therapeutic interventions

**Figure 1.**
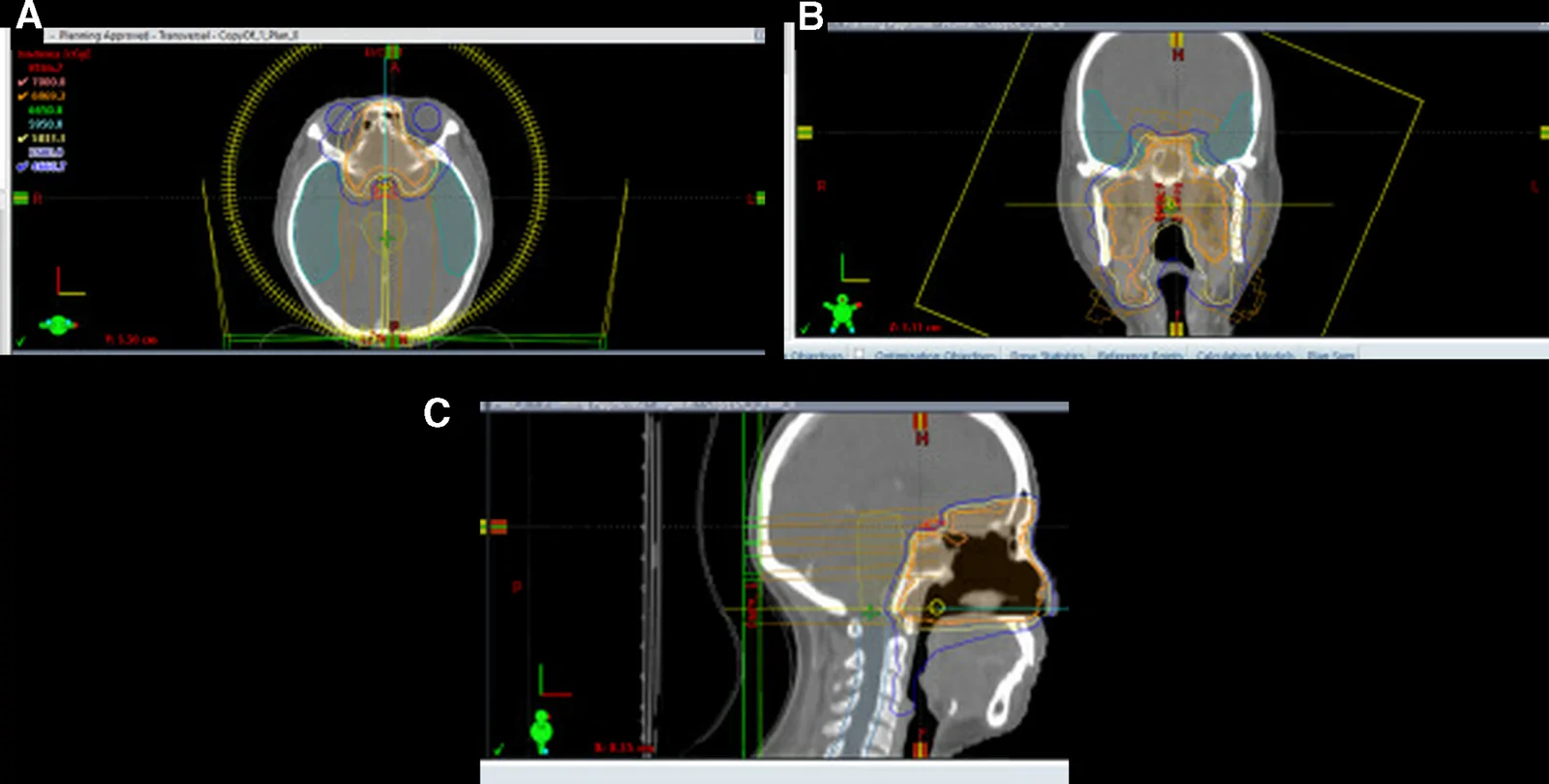
Postoperative VMAT/IMRT plan. (A–C) Representative axial, coronal, and sagittal planning CT images demonstrate target contours and dose distribution for the postoperative sinonasal and skull-base-related treatment volume. The final prescription was 70 Gy in 35 fractions. CT = computed tomography, IMRT = intensity-modulated radiotherapy, VMAT = volumetric modulated arc therapy.

First radiation-containing treatment phase. According to the radiotherapy prescription and planning record, postoperative volumetric modulated arc therapy/intensity-modulated radiotherapy (VMAT/IMRT) was delivered from November 3 to December 22, 2023. The clinical target volume included postoperative sinonasal and skull-base-related regions, the nasopharynx and bilateral parapharyngeal spaces, and bilateral cervical lymphatic drainage; the planning target volume (PTV) was generated with a 3-mm margin. The initial 50 Gy in 25 fractions was extended to a final prescription of 70 Gy in 35 fractions. Cisplatin 130 mg, divided over 2 days, was administered on November 7 and November 28, 2023. The plan is shown in Figure 1.

During treatment, headache intensity decreased from 8/10 to 2.5/10 on a patient-reported visual analog scale. Clinically recorded Snellen visual acuity improved from 20/200 to 20/50. A formal ophthalmologic examination report documenting this assessment was not available in the records accessible for review. Later in the course, worsening headache, visual symptoms, lumbar and radicular pain, lower abdominal pain, bilateral lower-limb paralysis, and urinary retention accompanied disseminated progression.

**Figure 2.**
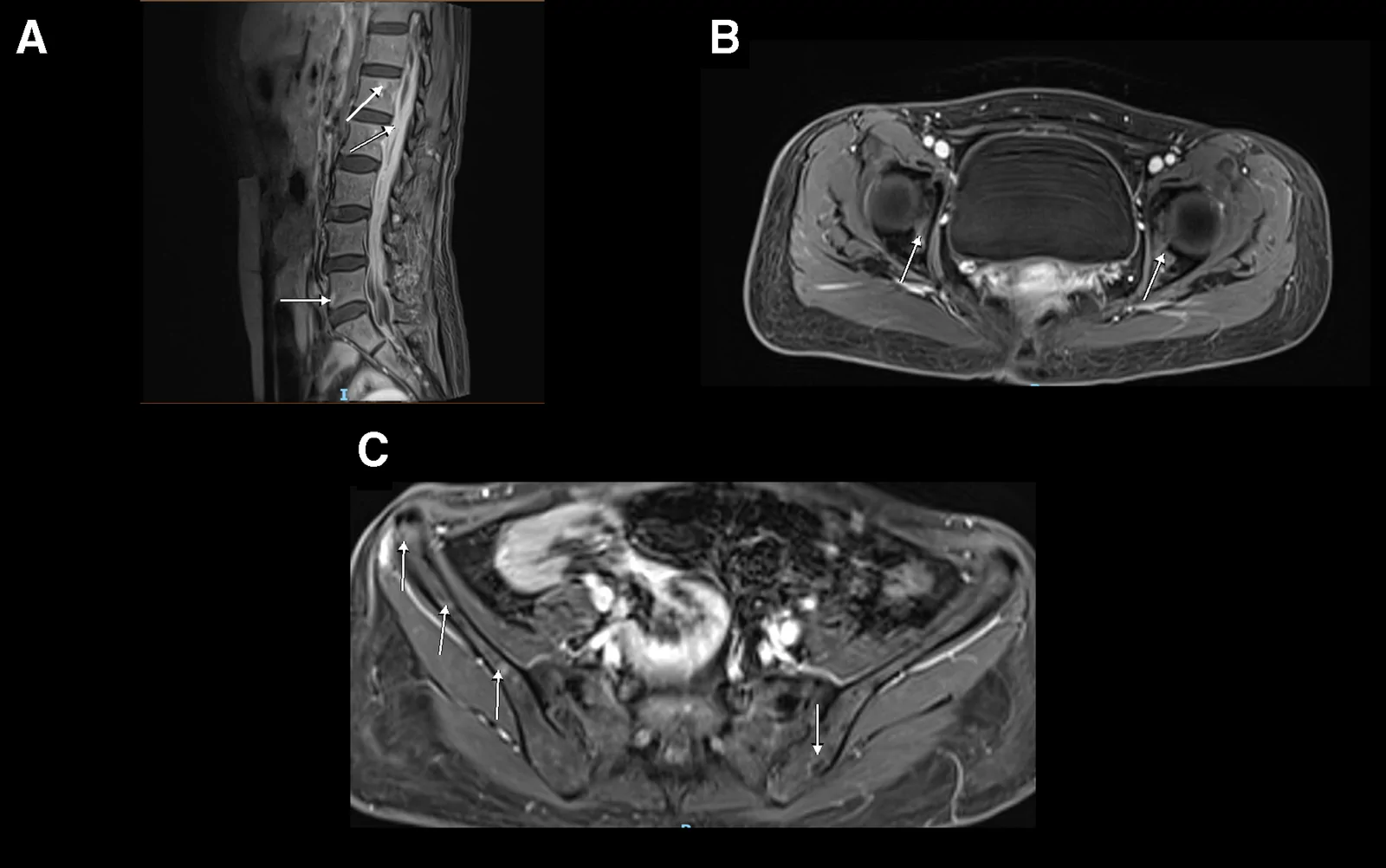
MRI findings before craniospinal irradiation. (A) Sagittal contrast-enhanced lumbar MRI obtained on January 8, 2024, demonstrates representative enhancing vertebral lesions at L1 and L5 and abnormal enhancement involving the filum terminale and cauda equina (arrows). (B) Axial contrast-enhanced pelvic MRI obtained on January 4, 2024, demonstrates representative bilateral acetabular lesions (arrows). (C) Axial contrast-enhanced pelvic MRI from the same examination demonstrates representative bilateral iliac lesions (arrows). The arrows identify representative abnormalities and are not intended to indicate the complete extent of disease. MRI = magnetic resonance imaging.

Second radiation-containing treatment phase. MRI examinations on January 4 and January 8, 2024, together with clinical assessment, demonstrated leptomeningeal disease involving the filum terminale and cauda equina, together with extensive osseous disease involving the lumbar spine, sacrum, bilateral ilia, acetabular regions, and left pubis (Fig. 2). Pembrolizumab 200 mg was administered on January 9, 2024.

**Figure 3.**
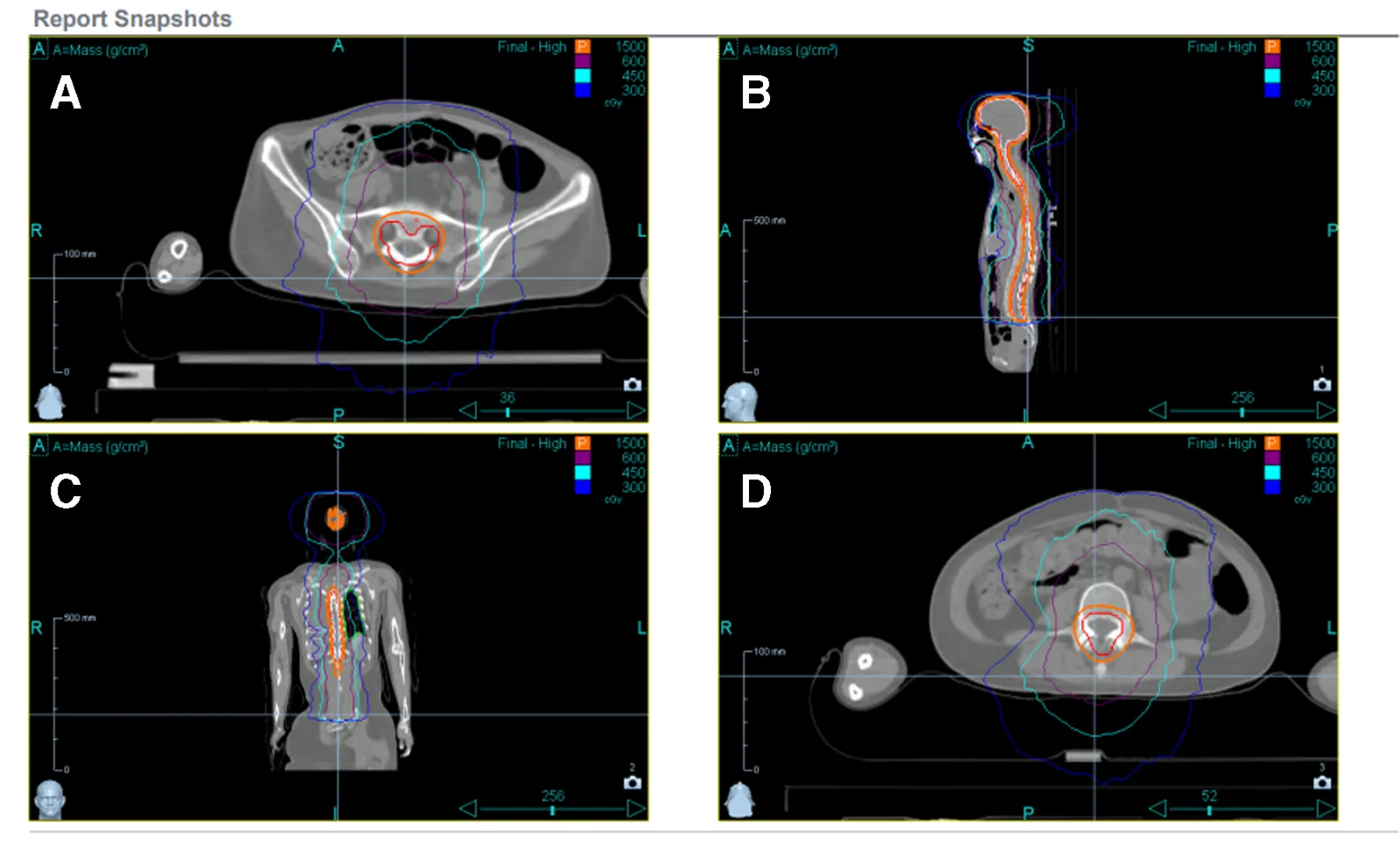
Palliative craniospinal irradiation plan. Representative axial (A, D), sagittal (B), and coronal (C) planning images demonstrate the craniospinal planning target volume and dose distribution. Helical IMRT/tomotherapy delivered 15 Gy in 10 fractions. IMRT = intensity-modulated radiotherapy, PTV = planning target volume.

Given the rapidly progressive disseminated disease, severe neurologic impairment, extensive metastatic burden, and limited expected prognosis, craniospinal irradiation was delivered with palliative intent at 15 Gy in 10 fractions to balance symptom relief, treatment burden, and normal-tissue exposure. Treatment was delivered from January 18 to January 31, 2024, using helical IMRT/Tomotherapy (Fig. 3). The PTV was approximately 2092.47 cm^3^, the mean PTV dose was approximately 15.68 Gy, and the recorded PTV coverage was approximately 99.99%. Pain decreased after CSI, whereas bilateral lower-limb paralysis persisted. Bevacizumab 500 mg and oral temozolomide were used after CSI, and pembrolizumab and bevacizumab were continued during subsequent care.

**Figure 4.**
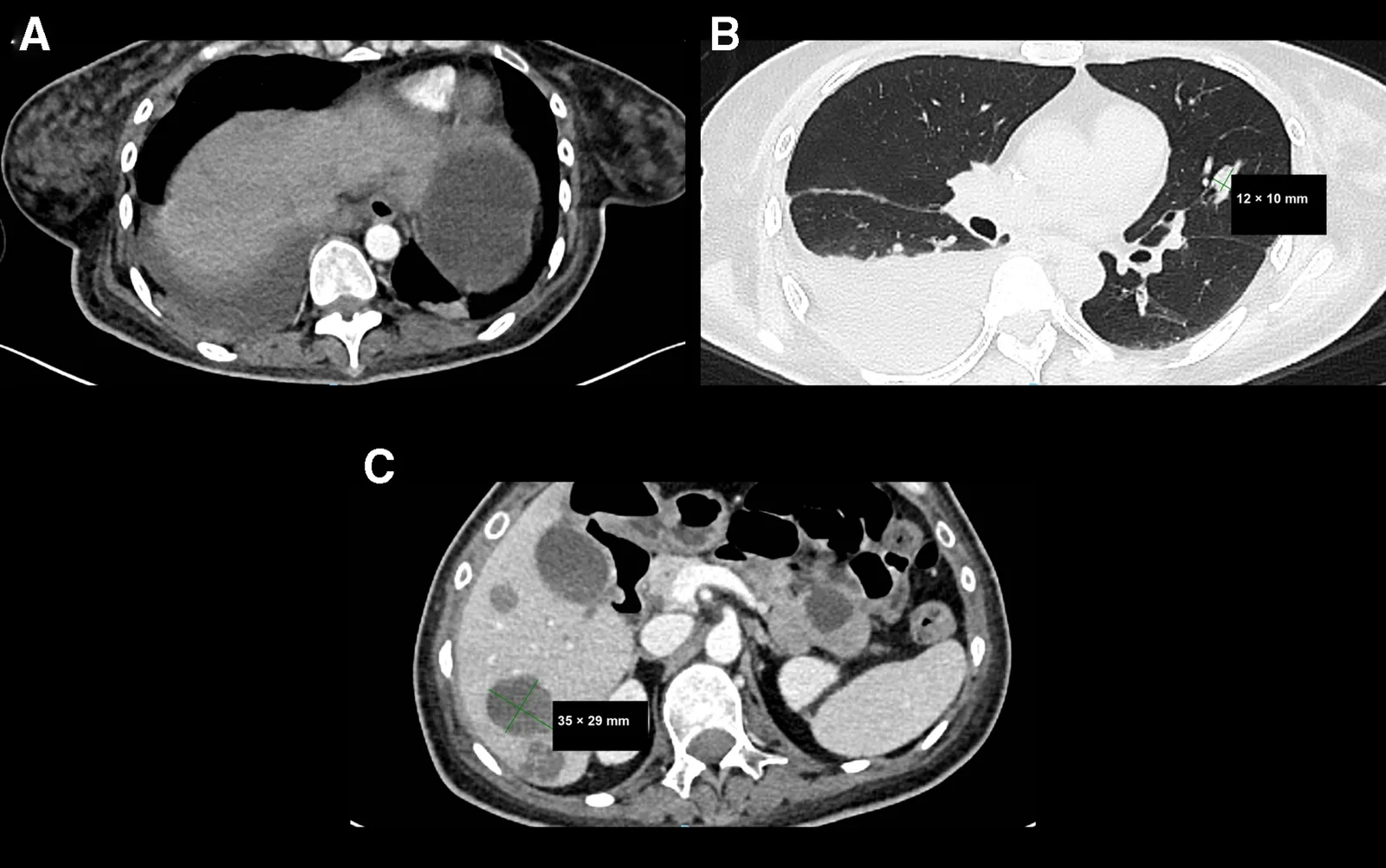
Thoracic and abdominal metastatic disease on CT obtained on March 11, 2024. (A) Large right pleural effusion with adjacent compressive atelectatic change. (B) A left pulmonary nodule measuring approximately 12 × 10 mm. (C) Multiple hepatic lesions, including a representative lesion measuring approximately 35 × 29 mm. These measurements represent disease burden on this examination and were not used as paired treatment-response measurements. CT = computed tomography.

Third radiation-containing treatment phase. In March 2024, the patient developed chest tightness and exertional dyspnea. Right pleural effusion was drained, and malignant cells were identified in the pleural fluid. CT on March 11 showed right pleural disease and effusion, a left lung nodule of approximately 12 × 10 mm, and multiple hepatic metastases, including a representative lesion of approximately 35 × 29 mm (Fig. 4). Intrapleural cisplatin 30 mg plus bevacizumab 300 mg was administered on March 18. Bevacizumab 500 mg was administered on March 26 and April 24, and pembrolizumab 200 mg on April 2 and April 23; oral temozolomide was used during this period.

**Figure 5.**
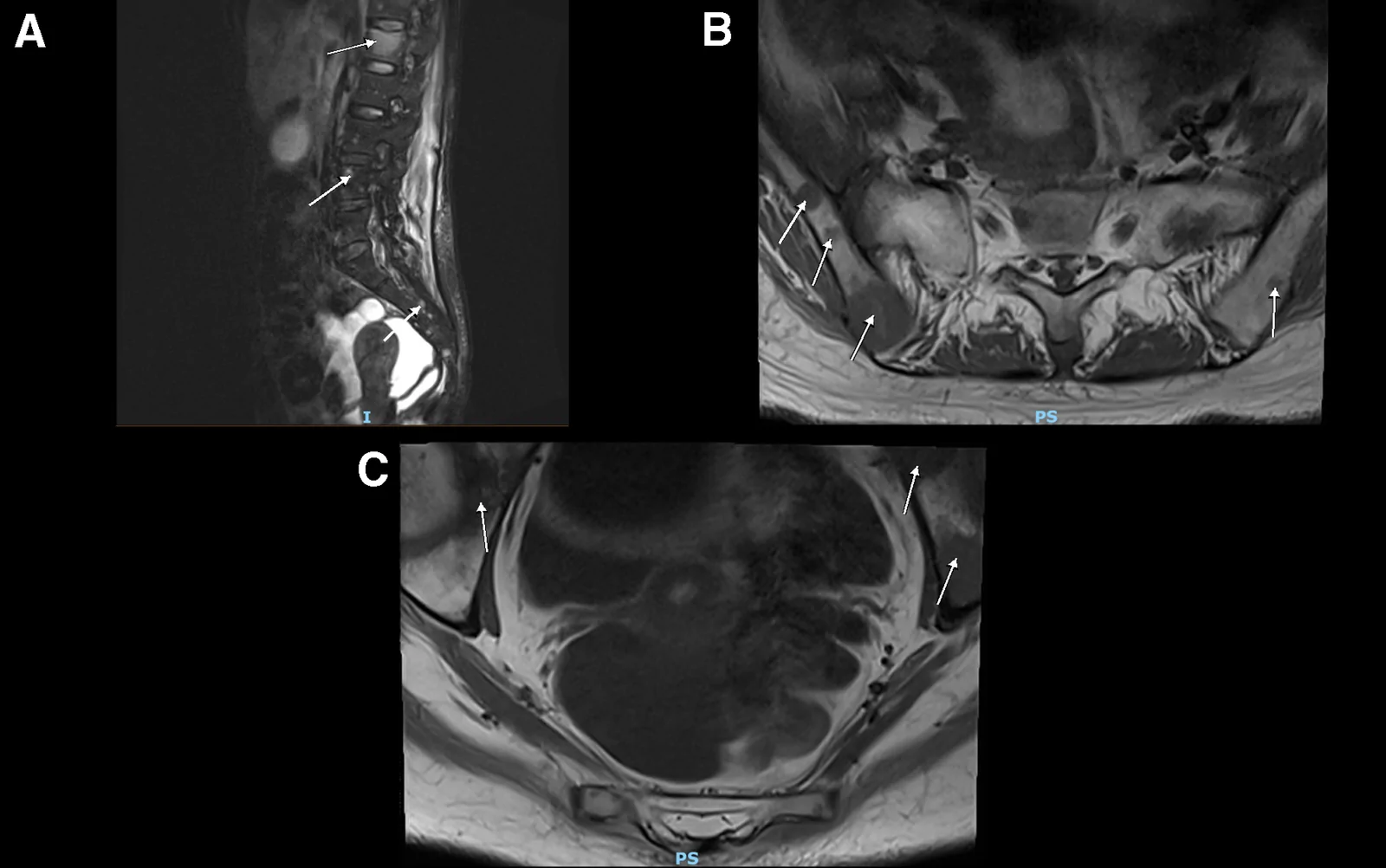
MRI findings before the third radiotherapy course. (A) Sagittal fat-suppressed T2-weighted MRI obtained on April 22, 2024, demonstrates representative abnormal marrow-signal lesions at L1, L4, and S3 (arrows). (B) Axial pelvic MRI demonstrates representative bilateral iliac marrow-signal abnormalities (arrows). (C) Axial pelvic MRI demonstrates representative bilateral acetabular marrow-signal abnormalities (arrows). The arrows identify representative abnormalities and are not intended to indicate the complete extent of osseous involvement. MRI = magnetic resonance imaging.

**Figure 6.**
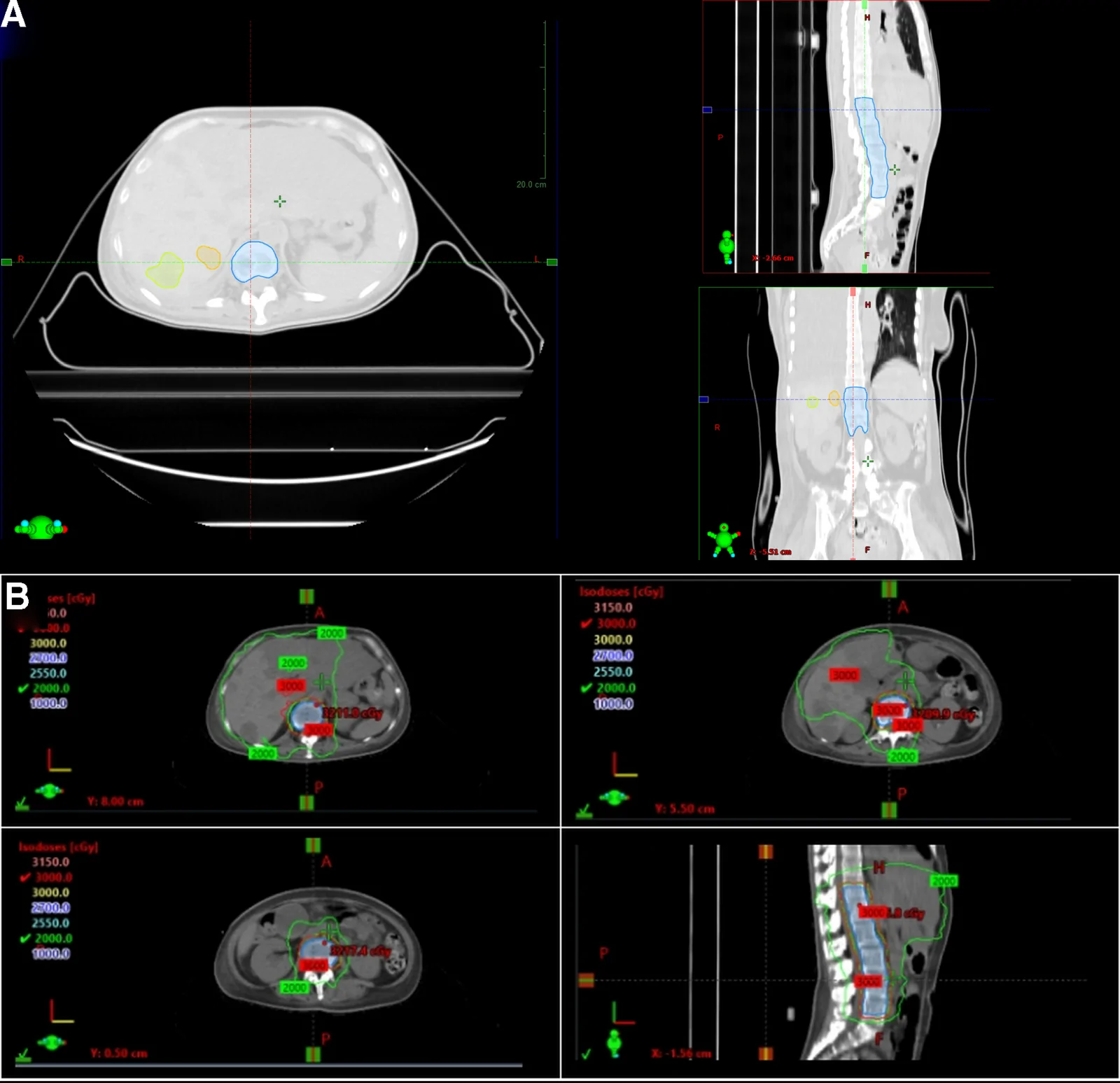
Target delineation and dose distribution for the third radiotherapy course. (A) Axial, sagittal, and coronal planning CT images demonstrate one lumbar metastatic target (blue contour) and 2 hepatic metastatic targets (green and yellow contours). (B) Representative axial and sagittal dose-distribution images demonstrate coverage of the 3 targets. All targets received 30 Gy in 10 fractions using a single VMAT plan. CT = computed tomography, VMAT = volumetric modulated arc therapy.

MRI on April 22, 2024, demonstrated multifocal abnormalities involving the lumbar spine, sacrum, bilateral ilia, acetabular regions, and right ischium in the setting of progressive lumbosacral pain. Representative images are shown in Figure 5. A single VMAT plan treated one lumbar metastatic target and 2 hepatic metastatic targets from April 28 to May 13, 2024; each target received 30 Gy in 10 fractions (Fig. 6). Lumbar pain decreased. Recurrent dyspnea during this period was managed with repeat pleural drainage, and the subsequent respiratory improvement was attributed to drainage and pleural management rather than to this radiotherapy course.

### 2.5. Follow-up and outcomes

Serial imaging assessments were performed throughout the clinical course and were contemporaneously interpreted by the treating multidisciplinary team as indicating interval reduction in selected lesions after the radiation-containing multimodal treatment phases. Because several examinations were performed at outside institutions or were no longer retrievable, uniform retrospective lesion-by-lesion remeasurement was not feasible. These observations were therefore interpreted as treatment-associated responses within multimodal care rather than effects attributable to radiotherapy alone.

According to telephone follow-up with the patient’s family, the patient died approximately 12 months after surgery.

## 3. Discussion

This report documents repeated clinical and imaging responses after 3 multimodal treatment phases incorporating radiotherapy. The first phase was postoperative chemoradiotherapy, the second was palliative CSI for neuroaxis and widespread osseous dissemination, and the third was palliative irradiation of one lumbar and 2 hepatic targets. The patient nevertheless developed rapid systemic progression and died approximately 12 months after surgery. Thus, the observations represent symptom relief and possible local treatment benefit, not durable disease control or survival benefit.

Published evidence is heterogeneous. Retrospective and pooled reports suggest that surgery and radiotherapy-based local management may be associated with better outcomes in selected head and neck NUT carcinoma cases, but selection bias and treatment heterogeneity limit causal inference.^[2,3,5]^ Individual reports describe complete or prolonged responses after concurrent chemoradiotherapy or multimodal therapy, whereas other cases progress rapidly despite intensive treatment.^[7–10]^

Muramatsu et al reported a complete response after concurrent chemoradiotherapy, Geng et al described prolonged disease control after chemoradiotherapy followed by consolidation systemic therapy, and Rijken et al reported durable remission after surgery, high-dose radiotherapy, and chemotherapy.^[7,9,10]^ In contrast, the present patient experienced repeated but transient clinical and imaging responses during radiotherapy-containing multimodal treatment, followed by rapid neuroaxis and systemic dissemination. This comparison underscores the distinction between phase-specific palliative benefit and durable disease control: repeated short-term responses may be clinically meaningful without implying prolonged control or survival benefit.

Response attribution must remain conservative. Cisplatin was administered with the first course; pembrolizumab preceded CSI; bevacizumab, pembrolizumab, and temozolomide were used during later periods; and pleural drainage and intrapleural treatment directly affected respiratory symptoms. Because systemic therapies were administered during overlapping periods, the independent contribution of radiotherapy could not be isolated. In particular, improvement in dyspnea followed drainage and was not attributed to the third radiotherapy course.

Radiotherapy can interact biologically with chemotherapy and immune therapy, including through DNA damage and immunogenic cell-death pathways, but such mechanisms remain hypotheses in this patient because no functional experiments were performed.^[11,12]^ Future reports should combine complete molecular characterization with prespecified imaging time points, standardized response assessment, and detailed dosimetry.

## 4. Limitations

This report has several limitations. First, it describes one patient and cannot establish causality, generalized intrinsic radiosensitivity, durable control, or survival benefit. Second, all radiation courses occurred within multimodal care, so the effects of radiotherapy cannot be separated from systemic and supportive treatments. Third, although serial imaging informed contemporaneous clinical decisions and showed interval changes, several outside or historical examinations could not be retrieved for uniform retrospective lesion-by-lesion remeasurement. Fourth, the original external molecular report was unavailable, precluding independent verification of the NUTM1 assay platform and fusion partner. Fifth, the source materials did not include a formal ophthalmology report for the recorded Snellen measurements. These limitations require conservative interpretation.

## 5. Conclusion

Repeated clinical and imaging responses were observed following multiple phases of multimodal treatment incorporating radiotherapy. Radiotherapy may provide clinically meaningful local or palliative benefit as part of individualized multimodal treatment in selected patients with NUT carcinoma. This single case does not establish radiotherapy-specific sensitivity, durable disease control, or a survival benefit.

## 6. Patient perspective

Because the patient died before manuscript revision, a formal first-person patient perspective could not be obtained for inclusion in this report.

## Author contributions

**Conceptualization:** Zhongqiu Wang.

**Data curation:** Qi An, Yuxuan Tao.

**Investigation:** Qi An, Yuxuan Tao, Peiguo Wang.

**Methodology:** Zhongqiu Wang.

**Project administration:** Peiguo Wang, Zhongqiu Wang.

**Resources:** Yuxuan Tao, Peiguo Wang.

**Supervision:** Zhongqiu Wang.

**Validation:** Yuxuan Tao, Peiguo Wang, Zhongqiu Wang.

**Visualization:** Qi An.

**Writing – original draft:** Qi An.

**Writing – review & editing:** Qi An, Yuxuan Tao, Peiguo Wang, Zhongqiu Wang.
